# Superbugs or multidrug resistant microbes: A new threat to the society

**DOI:** 10.1002/hsr2.1480

**Published:** 2023-08-02

**Authors:** Sakshi Painuli, Prabhakar Semwal, Rohit Sharma, Shopnil Akash

**Affiliations:** ^1^ Natural Products Research Laboratory Uttarakhand Council for Biotechnology Dehradun India; ^2^ Department of Biotechnology Graphic Era (Deemed to be University) Dehradun India; ^3^ Research and Development Cell Graphic Era Hill University Dehradun India; ^4^ Department of Rasa Shastra and Bhaishajya Kalpana Faculty of Ayurveda, Institute of Medical Sciences, Banaras Hindu University Uttar Pradesh Varanasi India; ^5^ Faculty of Allied Health Science Department of Pharmacy, Daffodil International University Dhaka Bangladesh

## AUTHOR CONTRIBUTIONS

**Sakshi Painuli**: Conceptualization; data curation; formal analysis; software; validation; visualization; writing—original draft; writing—review and editing. **Prabhakar Semwal**: Formal analysis; investigation; supervision; validation; visualization; writing—original draft; writing—review and editing. **Rohit Sharma**: Data curation; supervision; validation; visualization; writing—review and editing. **Shopnil Akash**: Validation; visualization; writing—review and editing.

## CONFLICT OF INTEREST STATEMENT

The authors declare no conflict of interest.

Dear Editor,

1

As the world struggles to recover from the recent COVID pandemic, antimicrobial resistance (AMR) is proving to be a new threat to human health, food security, and economic development. According to the World Health Organization (WHO), multidrug‐resistant pathogens/superbugs are considered a serious threat to health, worldwide. The Interagency Coordinating Group on AMR has estimated that by 2050, the mortality rate due to AMR may increase, with an estimated annual death rate of approximately 10 million people. The term multidrug resistant pathogens refers to those bacteria, viruses, fungi, or parasites that become resistant to the standard treatment and no longer respond to the treatment due to inappropriate use of antimicrobial drugs, consumption of unhygienic food, poor sanitary conditions, inappropriate infection prevention, and so forth.^1^ The rapid emergence of newly evolved resistance mechanisms and the low efficacy of drugs/treatments against repeated microbial infections lead to a prolonged illness, high health care costs, worsening disease, and an increased risk of death. In the last few decades, most of the infectious agents such as viruses, bacteria, fungi, and parasites have developed high levels of resistance with high morbidity and mortality rate and are therefore referred to as “superbugs.”

Usually, superbugs or multidrug resistance developed by natural phenomenon, however the morbidity rate increases in immunodeficient conditions, like diabetes, severe burn patients, HIV infection, COVID infection, organ transplant recipients, and so forth. According to a list published by WHO several types of bacteria are rapidly developing resistance to antibiotics and these bacteria have been categorized into three priority groups including *Acinetobacter baumannii* (carbapenem resistant), *Pseudomonas aeruginosa* (carbapenem resistant), Enterobacteriaceae (carbapenem resistant), and Extended‐spectrum beta‐lactamases producing bacteria as priority 1 pathogens; *Neisseria gonorrhoeae* (cephalosporin and fluoroquinolone resistant), *Enterococcus faecium* (vancomycin resistant), nontyphoidal *Salmonella* (fluoroquinolones resistant), *Staphylococcus aureus* (methicillin and vancomycin resistant), *Helicobacter pylori* (clarithromycin resistant), and *Campylobacter* spp. (fluoroquinolone resistant) as priority 2 pathogens; and *Haemophilus influenzae* (ampicillin resistant), *Shigella* species (fluoroquinolones resistant) and *Streptococcus pneumoniae* (penicillin resistant) as priority 3 pathogens (https://www.who.int/en/news-room/detail/27-02-2017-who-publishes-list-of-bacteria-for-which-new-antibiotics-are-urgently-needed; Accessed on 07/Nov/2022). Along with them, *Klebsiella pneumoniae* (carbapenems and cephalosporin resistant), *Escherichia coli* (fluoroquinolones and cephalosporin resistant), and *Mycobacterium tuberculosis* (fluoroquinolone, rifampicin, and isoniazid resistant), showed the highest resistant rates. Infections caused by fungal growth are treated with antifungal drugs such as Deoxyribonucleic and Ribonucleic acid synthesis inhibitors (flucytosine), azole derivatives (itraconazole, voriconazole, ketoconazole, and fluconazole), 1,3‐β‐glucan synthase inhibitors (echinocandins), and polyene macrolides (amphotericin B), although fungal strains like *Trichosporon beigelii*, *Aspergillus* spp., *Cryptococcus neoformans*, *Candida* spp*., Scopulariopsis* spp., and so forth, have gained resistance against these drugs.^2^ Parasitic pathogens such as Leishmania, Entamoeba, Plasmodia, *Toxoplasma gondiischi*, and *Trichomonas vaginalis* have developed resistance against drugs like amphotericin B, artemisinin, miltefosine, paromomycin, chloroquine, and pyrimethamine.^1^ Due to extensive use of drugs and constant viral replication, many viruses have also developed drug resistance over the years, including herpes simplex virus, hepatitis B and C virus, influenza A virus, HIV, cytomegalovirus, varicella‐zoster virus, SARS‐CoV‐2, and so forth, raising the concern of antiviral resistance in immunocompromised patients.^3^, ^4^, ^5^, ^6^ These superbugs are capable of causing a high percentage of hospital acquired infections and can turn common infections such as pneumonia, bloodstream infections, urinary tract infections, and so forth, into fatal diseases and can be deadly to COVID patients.

The COVID‐19 pandemic, which spread uncontrollably in different parts of the world, has reported up to 532 million positive cases, including over 6 million deaths worldwide, till June 2022.^7^ Although, it seems like we are winning the battle against COVID, it is believed that in the future we will have to face different health related issues caused by this pandemic. One of the biggest issues is excessive consumption of antimicrobial drugs against COVID has increased, which has now rapidly increased the infection rate of multidrug resistant related diseases.^8^, ^9^, ^10^ In some mild cases of COVID‐19, it has been observed that there is an increase in the vulnerability of patients to serious illness due to the co‐infection with multidrug resistant pathogenic microbes.^11^, ^12^ Posterarao et al.^13^ reported that COVID patients with diabetes mellitus (type 2) suffered from serious infections by *Candida glabrata* and other fungi. Another report suggests that the fungal strain *Aspergillus fumigatus* worsened the condition of patients suffering from acute COVID infection.^14^ Cebeci Kahraman et al.^15^ reported the cutaneous and systemic adverse reactions in the vaccinated COVID patients from the Turkey region. The study included 2290 COVID patients vaccinated by Pfizer/BioNTech (BNT162b2) and Sinovac/CoronaVac (inactivated SARS‐CoV2) vaccines, and the result showed that serious reactions like herpes zoster, anaphylaxis, and triggered autoimmunity appeared in the patients vaccinated from CoronaVac vaccine.^15^ In another study Vinayagamoorthy et al.^16^ revealed that the invasive medical measures and disease itself in COVID‐19 patients make them more susceptible to the infection of multidrug resistant *Candida auris*.

Although several new techniques and approaches including bio‐nanotechnology approaches, antimicrobial polymeric biomaterials products, combinational drug approaches, and so forth, have been developed in recent years to combat various AMR.^17^ Natural products have been extensively researched against AMR pathogens. Geraniol, neral, 1,8‐cineole, camphene, alpha‐curcumene and beta‐phellandrene isolated from *Zingiber officinale* have shown antibacterial properties against 18 resistant pathogens.^18^ Lariciresinol and berberine of *Z. officinale* showed significant activity against *Salmonella enterica serovar typhimurium*.^19^ Phenols and flavonoids obtained from the *Vernonia auriculifera* Hiern have shown activity against *E. coli, Enterobacter aerogenes, K. pneumoniae, P. aeruginosa* and *S. aureus*.^20^ Pleuromutilin, azamulin, valnemulin, tiamulin and reptapamulin have shown significant activity against Methicillin‐resistant *S. aureus*.^21^ In addition, metal and metal oxide nanoparticles have shown significant antibacterial activity. Aunkor et al.^22^ reported the antibacterial activity of graphene oxide nanosheet against multidrug resistant superbug and Roy et al.^23^ reported the prominent activity of photothermal therapy against superbugs. Zinc oxide nanoparticles prepared from fruits and leaves of *Limonia acidissima* have shown activity against *K. pneumonia*' *Shigella, Streptococus, staphylococcus* and *S. paratyphi*.^24^ Silver nanoparticles synthesized from *Aloe vera* also showed the antimicrobial activity against *S. aureus, E. coli, P. pseudomonas, A. baumannii* strains.^25^

Over time, there has been a sharp reduction in the emergence and approval of new antimicrobials. Dalbavancin, oritavancin, meropenem and vaborbactam, eravacycline, pretomanid, cefiderocol, plazomicin, delafloxacin and tedizolid are some of the recently approved antibiotics.^26^ The new antifungal drugs under development are fosmanogepix, tetrazoles (VT‐1129, 1161, and 1598), Aureobasidin A, F901318, T‐2307, VL‐2397.^27^ According to a report published by WHO in 2020 only 52 new antimicrobial compounds were under preclinical and clinical trials, even more terrifying is the fact that just 32 of these were approved for treating multidrug resistant pathogens.^28^ Cefiderocol, probenecid, durlobactam, murepavadin, cestobiprole, and so forth, are example of few antibiotic drugs which are effective against multidrug resistant pathogens.^17^

Presently, many drugs and vaccines for these resistant pathogens are under clinical trial, which are likely to be available in the upcoming years, but in the meanwhile with the help of some control and preventive measures, we can reduce the number of active cases of multidrug resistant pathogens. These preventive measures include proper hygiene practices, like appropriate sanitation and protective measures must be taken to avoid human to human transmission of resistant strains. Antimicrobial drugs should be prescribed judiciously and unnecessary length of treatment and use of broad‐spectrum antimicrobial drugs should also be avoided. The awareness of both the physician and the patient regarding extensive and inappropriate use of antimicrobial drugs can suppress the rise of AMR strains. Both developing and developed countries must follow certain rules and regulations to prevent wasteful drug marketing. The use of drugs against highly resistant microbial strains in feed for animals like chicken, pig, fish and so forth, should be investigated, as they can directly develop resistant strains in humans. One should consume good and healthy food which has high nutrient and vitamin content to boost immunity naturally.

In conclusion, as we know that the development of new and effective drugs, vaccines, and therapies against multiple drug resistant pathogens is in the pipeline. In the last few years, we have also seen the deadly consequences of COVID, from which we should take lessons and prepare ourselves for the upcoming threat of superbugs. The combinational drug approaches, nano‐particles based formulations, antimicrobial polymeric biomaterial products, new vaccine technology and so forth, can be good alternatives to treat multidrug resistant pathogens. Additionally, preventive measures, consuming good and healthy food which has high nutrient and vitamin content can help in combating against AMR.

## Data Availability

All data used to support the findings of this study are included in the article.
